# The impact of COVID-19 on student learning in New South Wales primary schools: an empirical study

**DOI:** 10.1007/s13384-021-00436-w

**Published:** 2021-03-12

**Authors:** Jennifer Gore, Leanne Fray, Andrew Miller, Jess Harris, Wendy Taggart

**Affiliations:** Teachers and Teaching Research Centre, School of Education, University of Newcastle, Newcastle, Australia

**Keywords:** Student outcomes, COVID-19, Pandemic, Public school, Primary education

## Abstract

The COVID-19 pandemic produced widespread disruption to schooling, impacting 90% of the world’s students and moving entire school systems to remote and online learning. In the state of New South Wales, Australia, most students engaged in learning from home for at least eight weeks, with subsequent individual and intermittent school closures. However, while numerous claims have circulated in the popular media and in think tank reports, internationally, about the negative impacts on learning, there is limited empirical evidence of decreased student achievement. Drawing on data from more than 4800 Year 3 and 4 students from 113 NSW government schools, this paper compares student achievement during 2019 and 2020 in a sample of matched schools to examine the effects of the system-wide disruption. Somewhat surprisingly, our analysis found no significant differences between 2019 and 2020 in student achievement growth as measured by progressive achievement tests in mathematics or reading. A more nuanced picture emerges when the sample is examined by dis/advantage (ICSEA) and Year level. The Year 3 cohort in the least advantaged schools (ICSEA < 950) achieved 2 months less growth in mathematics, while the Year 3 students in mid-ICSEA schools (950–1050) achieved 2 months’ additional growth. No significant differences were identified for Indigenous students or students located in regional locations. These results provide an important counter-narrative to widespread speculation about alarming levels of ‘learning loss’ for all students. While the lower achievement growth in mathematics for Year 3 students in lower ICSEA schools must be addressed as a matter of urgency to avoid further inequities, most students are, academically, where they are expected to be. Our findings are a testament to the dedicated work of teachers during the 2020 pandemic to ensure that learning for most students was not compromised, despite unusually trying circumstances.

## Introduction

The COVID-19 pandemic led to unprecedented disruption to schooling in more than 190 education systems globally, impacting more than 90% of the world’s school students (Psacharopoulos et al. 2020; UNESCO 2020a; United Nations 2020). In late-March 2020, throughout Australia, parents were urged to keep their children at home, resulting in a swift and dramatic shift from face-to-face learning to flexible and remote delivery of education. In New South Wales government schools, ‘learning from home’ continued for two months for most students, except for the children of essential workers who continued to attend school. Upon return to face-to-face teaching, many schools also closed intermittently for deep cleaning after students or teachers returned positive COVID-19 tests. In addition, extensive restrictions to usual school practices were mandated (NSW Department of Education 2020a), including the cancellation of school excursions, assemblies, sporting activities and large gatherings (Australian Government Department of Health 2020).

This widespread disruption to traditional teaching has raised concerns, globally, that student learning has been substantially negatively impacted as teachers, school leaders and students navigated online education (Burgess and Sievertsen 2020; Hampshire 2020; Joseph and Fahey 2020). While the shift to online schooling was promoted as a key way to support continuous learning in such crisis conditions (Baytiyeh 2019), schools and teachers were required to implement online learning in a matter of days, developing their knowledge and skills for teaching in remote and flexible contexts with minimal professional development (Clinton 2020) and, arguably, at unreasonable speed (Norman 2020; Potts Rosevear 2020). At the same time, students faced a range of environmental barriers and enablers to learning. These included varying levels of parental supervision, and differing access to the internet and devices required to sustain their learning (Burgess and Sievertsen 2020; CIRES and Mitchell Institute 2020; Engzell et al. 2020). Of particular concern was how to support already vulnerable and disadvantaged students trying to ‘learn from home’ (Gulosino and Miron 2017).

This ‘quarantine recess’ (Hinson et al. 2007) from traditional schooling generated substantial negative commentary about short-term and long-term effects on student outcomes and well-being, as well as the morale, self-efficacy and skills of teachers. While some commentators argued that a significant break from schooling does not necessarily have long-term effects on student learning outcomes (Hattie 2020), others invoked evidence that such breaks may result in student regression in basic skills and learning (Ofsted 2020a), increased disengagement and higher levels of student attrition (Brown et al. 2020). Indeed, recent reports predict that this period of school closure and shift to online learning could lead to poorer educational outcomes for almost 50% of Australian students (Brown et al. 2020; Finkel 2020), and not just in the short term (United Nations 2020).

However, to date, there remains limited robust empirical evidence about the extent to which students have been affected by the system-wide movement to online and remote learning. This is understandable, given the recent moratorium in Australia on NAPLAN—Australia’s major annual source of comparative achievement data. Other forms of testing have been implemented, at the school and state level, but their validity and reliability have not been established, especially when there are no directly comparable data from the start of the school year or previous cohorts.

Empirical evidence of the actual impact of the pandemic on student learning around the world has also been scarce, with just a handful of studies emerging in November and December 2020, none peer reviewed. Ofsted (2020a, b) reported, after visiting and talking with staff at 380 schools, that children of all ages in the United Kingdom lost some learning and basic skills. In the United States, Dorn et al. (2020) reported that elementary school students beginning the 2020–2021 school year were starting school, on average, 3 months behind in mathematics and one and a half months behind in reading compared with earlier cohorts (Dorn et al. 2020). A study using national standardised test data collected just prior to and just after an eight-week period of closedown in the Netherlands concluded that students lost one-fifth of a year’s learning having made little or no progress while learning from home (Engzell et al. 2020). In December, the NSW Department of Education reported results from *Check-in* assessments in reading and numeracy. More than 62,000 Year 3 students (or 88% of all Year 3) from 1439 schools were tested during the end of Term 3 and beginning of Term 4. Year 3 students were found to be on their expected trajectory for numeracy, but three to four months behind their expected trajectory in reading (NSW Department of Education 2020b).

To date, estimation and speculation have been the main drivers of debate and policy. For example, in Australia, influential modelling by the Grattan Institute (Sonnemann and Goss 2020) predicted a learning loss of 1 month from a two-month period of school disruption for the most disadvantaged students. However, valid inference requires data from before and after school closedown and a relevant comparison group (Engzell et al. 2020). Our study provides a comprehensive analysis of comparable data drawn from students in 2019 and 2020. In so doing, we offer insights for policy and practice by demonstrating, for this cohort at least, what actually happened during the widespread disruption to schooling-as-usual.

Rigorous empirical evidence is critical as a responsible basis for strategic action to address the effects of the quarantine recess on students and teachers. Without such evidence, school systems globally are relying on a small body of literature that focuses primarily on internal school and system crises such as school shootings (Thompson et al. 2017) and environmental disasters including fires, hurricanes, earthquakes and tornadoes. Much of this research focuses on individual school closures (Alvarez 2010; Convery et al. 2010; Ho et al. 2012; Trethowan and Nursey 2015) rather than the recent system-wide transition to online learning, an unprecedented occurrence. While the extant literature provides an important context for understanding the effects of crises and disasters on school leaders, teachers, students and the broader school community, it is severely limited in its capacity to inform schools and school systems in the transition back from learning at home following a system-wide period of school closure.

When the global impacts of the COVID-19 pandemic were beginning to become apparent, UNESCO (2020b) released a report outlining how the pandemic could be used to improve schooling and make education systems more inclusive; to “build back better” (para. 10). Despite this worthy manifesto, prior research on schooling following natural or other disasters suggests that such disruptions tend to exacerbate and highlight existing inequities rather than generate insights that repair them (Carr-Chellman et al. 2008; Ezaki 2018). The design of our study allows for fine-grained analysis of outcomes in relation to school-level dis/advantage. Specifically, we draw on comparable student achievement data from the school year prior to COVID-19 to examine the effects of this rapid system-wide change on student learning outcomes.

We did not set out to study the effects of COVID-19. Instead, we were in the middle of a randomised controlled trial (RCT) on the effects of Quality Teaching Rounds professional development, split across 2019 and 2020 cohorts. The Australian school year starts in late January and concludes in late December, which aligns annual student achievement testing with the calendar year—unlike in many other countries where the school year starts around August. Serendipitously, when COVID-19 struck, we had collected pre- and post-intervention data for 2019 and pre-intervention data from 2020 for most schools in the second cohort. The late-March closedown of schools in NSW meant we missed out on data collection in a small number of schools. The upside was that data collected just prior to the shutdown were comparable with data from the 2019 control group of schools.

Fortuitously, given the relatively low number of COVID-19 cases in Australia (at the time of writing 28,842 cases and 909 deaths), schools in NSW re-opened in plenty of time for follow-up data collection which commenced in late October and concluded in early December. Just when the worldwide crisis was worsening and schools were still shut down or shutting down in many parts of the world, we were able to re-purpose our 2020 baseline data and go back into schools to investigate effects of the pandemic on student learning.

## Methodology

In 2019, baseline (Term 1) and follow-up (Term 4) data were collected in 62 government schools for the *Building Capacity for Quality Teaching in Australian Schools* project. This group of schools formed the control group for an RCT examining the effects of a form of professional development, Quality Teaching Rounds (QTR), on student achievement (Gore et al. 2021; Miller et al. 2019). In 2020, equivalent data for a second cohort of 51 schools were collected in Term 1 (prior to the pandemic closure) as a part of the same RCT (which had to be postponed because of COVID-19) and gathered again at the end of the 2020 school year (Table 1) (post-pandemic closure). These data take the form of student achievement tests (Progressive Achievement Tests [PATs] in mathematics, reading and science) (Australian Council of Educational Research [ACER] 2011), and student surveys and teacher surveys as outlined below. Interviews were added for a subset of the 2020 teacher cohort to shed light on their experiences and perceptions of what happened for their students in terms of learning and well-being and what it was like to teach during this unusual year. In this first paper, we report on student achievement in mathematics and reading. Subsequent papers are currently in development focussing on the effect of learning from home on student well-being, teacher well-being, morale and self-efficacy.

**Table 1:** Data collection (2019–2020)

	Term 1 (Jan–Apr)	Term 2 (Apr–Jul)	Term 3 (Jul–Sep)	Term 4 (Oct–Dec)
Teachers				
2019	Survey	Survey	Survey	Survey
2020	Survey		Survey, interviews	Survey
Students				
2019	Survey, PATs			Survey, PATs
2020	Survey, PATs			Survey, PATs

### Student achievement

Students completed Progressive Achievement Tests (PATs) in mathematics, reading and science (Australian Council of Educational Research [ACER] 2011) in Term 1 and Term 4, 2020, administered by trained research assistants. The same data had been collected from students in Term 1 and Term 4, 2019.

### Instructional volume

The average time per week dedicated to each subject area was investigated using the teacher survey. Completed in Term 4, 2019 and at three time points in 2020 (Term 1, Term 3 and Term 4), teachers were asked “*How many hours a week on average do your students spend learning the following subjects (to the nearest hour): for numeracy (mathematics), literacy (reading), reading for comprehension, and science?*” Reading for comprehension was included as a subset of literacy because the reading test largely focuses on this capability.

### Sample

Students and teachers from 51 schools participated in the study during 2020. These data were compared with data collected from 62 public schools in 2019 for the *Building Capacity for Quality Teaching in Australian Schools* project. Schools that participated in 2019 were primarily located in major cities (*n* = 35) and regional areas (inner regional, *n* = 21; outer regional, *n* = 5). One school was in a very remote area. A similar pattern characterised schools that participated in 2020, with most in major cities (*n* = 40), and a smaller group in regional areas (inner regional, *n* = 10; outer regional, *n* = 1). There were no schools from remote or very remote communities in the 2020 sample (see Appendix).

Slightly more students completed achievement tests in 2019 (*n* = 2738) than in 2020 (*n* = 2156). The mean age of students in each cohort was 9.7 years and there were equal proportions of female participants (50%) and students from language backgrounds other than English (LBOTE) (24%) in both samples. Slightly more Indigenous students participated in 2019 (7%) than in 2020 (6%) (see Appendix).

We conducted a set of preliminary analyses using all of the data. However, to guard against cohort effects, or different starting points in student achievement, for the present analysis we drew on a sample of matched classes within schools (to account for in-school variance) from 2019 and 2020 for analysis. The procedure was designed to match a subset of schools on both baseline achievement and the socio-demographic variable of school ICSEA. Individual samples were created for Year 3 and Year 4 students. While mathematics and reading achievement are highly correlated in Years 3 and 4, science achievement is much more variable, and for the purpose of obtaining the closest baseline achievement match, science was dropped from this analysis. This process produced a total sample of 3030 students (1584 in 2019, and 1446 in 2020).

Classes within schools were ranked using the class level mean of the combined mathematics and reading percentile score at baseline (rounded to the nearest integer). Classes were ranked (ascending) by ICSEA and baseline achievement within ICSEA categories (low ≤ 950; mid = 950—1049; high = 1050 +). 2019 and 2020 classes within each one percentile block were paired with the closest ICSEA class if they were within ± 25 ICSEA. To retain as much data as possible, remaining 2020 classes were matched to 2019 classes that were within ± 2 percentile blocks and the closest ICSEA within ± 25 ICSEA. Sample characteristics of the matched subset of schools are provided in Table 2.

**Table 2:** Sample characteristics (2019, 2020)

	Year 3		Year 4		Total	
Characteristics	2019	2020	2019	2020	2019	2020
Schools, *n*	35	35	40	37	51	46
ICSEA, mean (SD)	992 (64)	996 (74)	1005 (71)	1000 (68)	1003 (70)	1003 (67)
ICSEA < 950, mean (SD)	918 (29)	916 (33)	916 (21)	912 (33)	914 (28)	917 (31)
ICSEA 950–1049, mean (SD)	993 (25)	1000 (25)	996 (27)	994 (25)	998 (28)	994 (27)
ICSEA 1050 +, mean (SD)	1099 (32)	1088 (27)	1106 (26)	1093 (30)	1103 (25)	1092 (27)
ICSEA < 950, *n* (%)	9 (26)	8 (2)	9 (23)	7 (19)	12 (24)	10 (22)
ICSEA 950–1049, *n* (%)	20 (57)	20 (57)	21 (53)	20 (57)	27 (53)	25 (54)
ICSEA 1050 +, *n* (%)	6 (17)	7 (20)	10 (25)	9 (24)	12 (24)	12 (26)
Regional, *n* (%)	17 (49)	10 (29)	18 (45)	7 (19)	23 (45)	10 (22)
Students, *n*	779	690	805	756	1584	1446
Age—years, mean (SD)	9.2 (0.5)	9.2 (0.5)	10.2 (0.4)	10.1 (0.4)	9.7 (0.6)	9.7 (0.7)
Female, *n* (%)	382 (49)	340 (49)	398 (49)	381 (50)	780 (49)	721 (50)
Indigenous, *n* (%)	72 (9)	60 (8)	32 (4)	38 (5)	104 (7)	98 (7)
LBOTE, *n* (%)	120 (15)	101 (15)	173 (22)	182 (24)	293 (19)	283 (20)

*ICSEA* Index of socio-educational advantage, *SD* standard deviation

### Analysis

Linear mixed models were fitted to compare continuous outcomes for each of the cohorts (2019 and 2020). Year (2019 and 2020), time (Baseline [Term 1] and follow-up [Term 4]) and year-by-time interactions were assessed as categorical fixed effects within the models. A repeated measures statement was included to model the within-subject correlated errors across time, and random intercepts were included for students within schools to account for the hierarchical nature of the data. Students who answered all questions correctly at the baseline assessment time-point were excluded from analysis as growth could not be assessed for these students. Differences of means and 95% confidence intervals (CIs) were determined using the linear mixed models, and the 2019 cohort was set as the comparison group for group-by-time contrasts.

Cohen's (1988) *d* was used to determine effect sizes (*d* = (Mchange2020 – Mchange2019)/*σ* pooled), where Mchange is the change in mean score for each group relative to their baseline value and *σ* is the pooled unconditional standard deviation. Ninety-five per cent confidence intervals (95% CIs) of the effect size were computed using the compute.es function (AC Del Re 2013) in R version 3.4.4 (R Core Team 2019). This function computes confidence intervals using the variance in *d* derived by the Hedges and Olkin (1985) formula.

Given widespread concern for less advantaged students, subgroup analysis was conducted to investigate if student outcomes differed across cohorts among ICSEA bands (low ≤ 950, mid = 950–1049 and high = 1050 +) or for Indigenous and regional students. As the comparison of growth between the two cohorts (year-by-time interaction) was the parameter of interest, the linear mixed models were repeated separately for each group within sub-groups (as opposed to running a three-way interaction term), using the entire student dataset.

The analysis is exploratory in nature; as such, no adjustments for multiplicity were applied to the group-by-time contrasts. However, we have provided footnotes on the impact of adjusting for multiple comparisons.

### Notes on interpreting the results

The following notes are designed to assist with interpretation of the results, especially for readers unfamiliar with the kinds of statistics used in the analysis. When viewing the PAT tables, the main columns to consider are the two on the right. Only those cells in the far-right column with an asterisk indicate a significant difference between the 2019 and 2020 cohorts. The second column from the right indicates the direction of the difference. Any effect size starting with a negative (e.g. − 0.12) indicates lower results for the 2020 cohort. Significant effects without a negative indicate greater growth for the 2020 cohort. Using standards adopted by the Education Endowment Foundation (EEF) (2018), effect sizes between 0.05 and 0.09 are equivalent to one month’s difference in growth while effect sizes between 0.10 and 0.18 indicate two months’ difference.

When viewing the figures, the bold lines indicate the trend for each cohort, showing the change from Term 1 to Term 4. They do not predict the variability underpinning the overall trend. However, it is not within the scope of this paper to explore these very interesting individual patterns.

## Results

The results are presented below with minimal commentary, which we provide in the discussion. In this section, we simply describe the findings.

### Student achievement in mathematics and reading

**Fig. 1:**
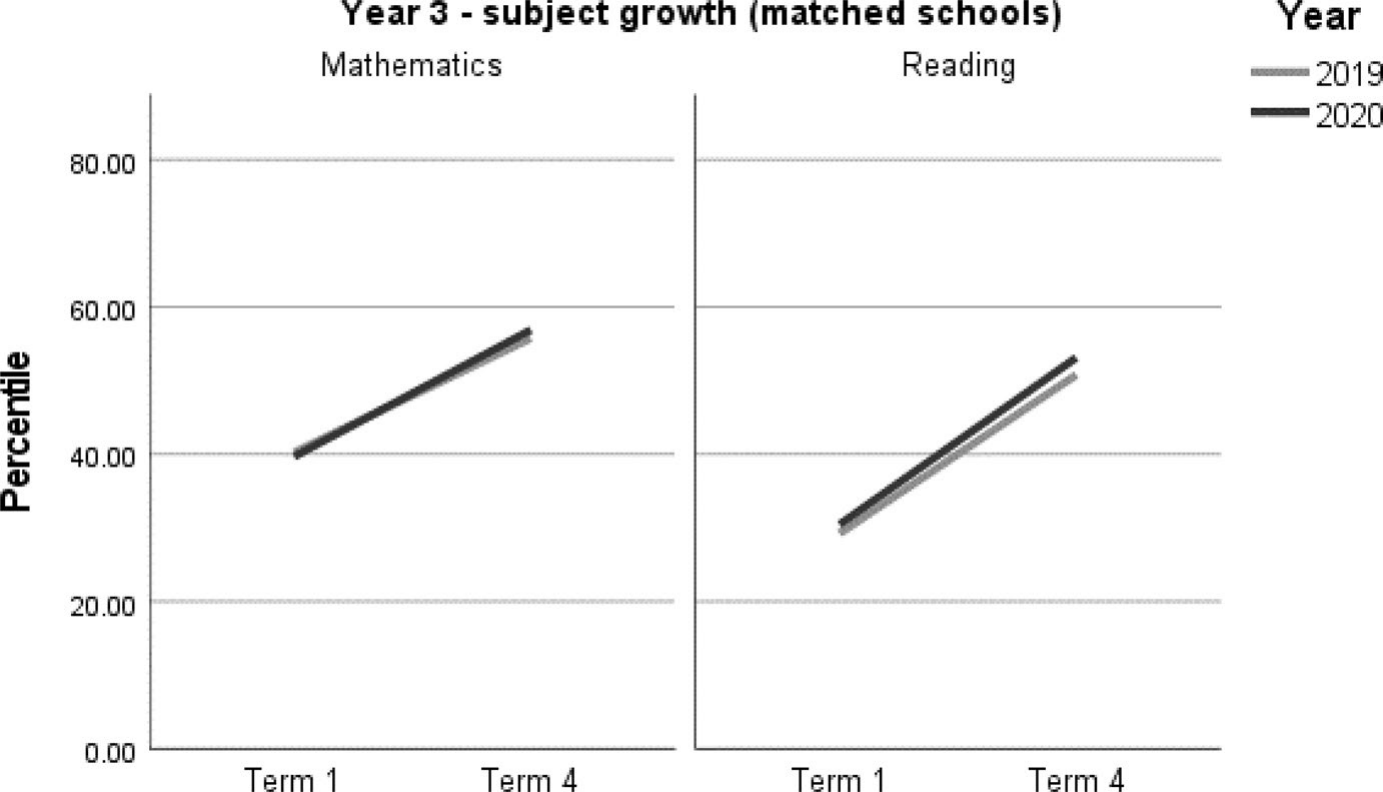
Year 3 student achievement growth in Mathematics and Reading (2019–2020)

**Fig. 2:**
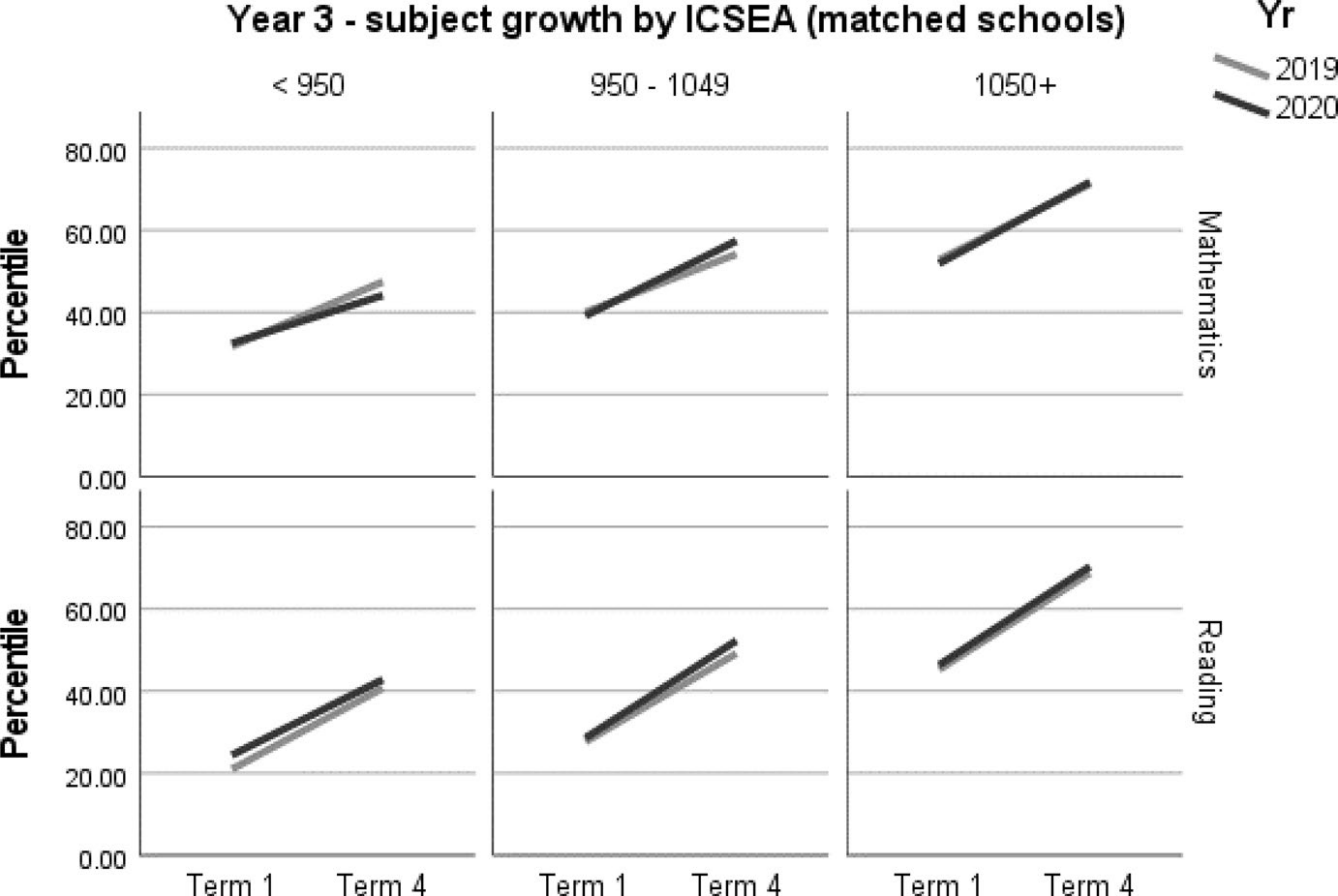
Year 3 student achievement in mathematics and reading (2019–2020) by ICSEA

**Fig. 3:**
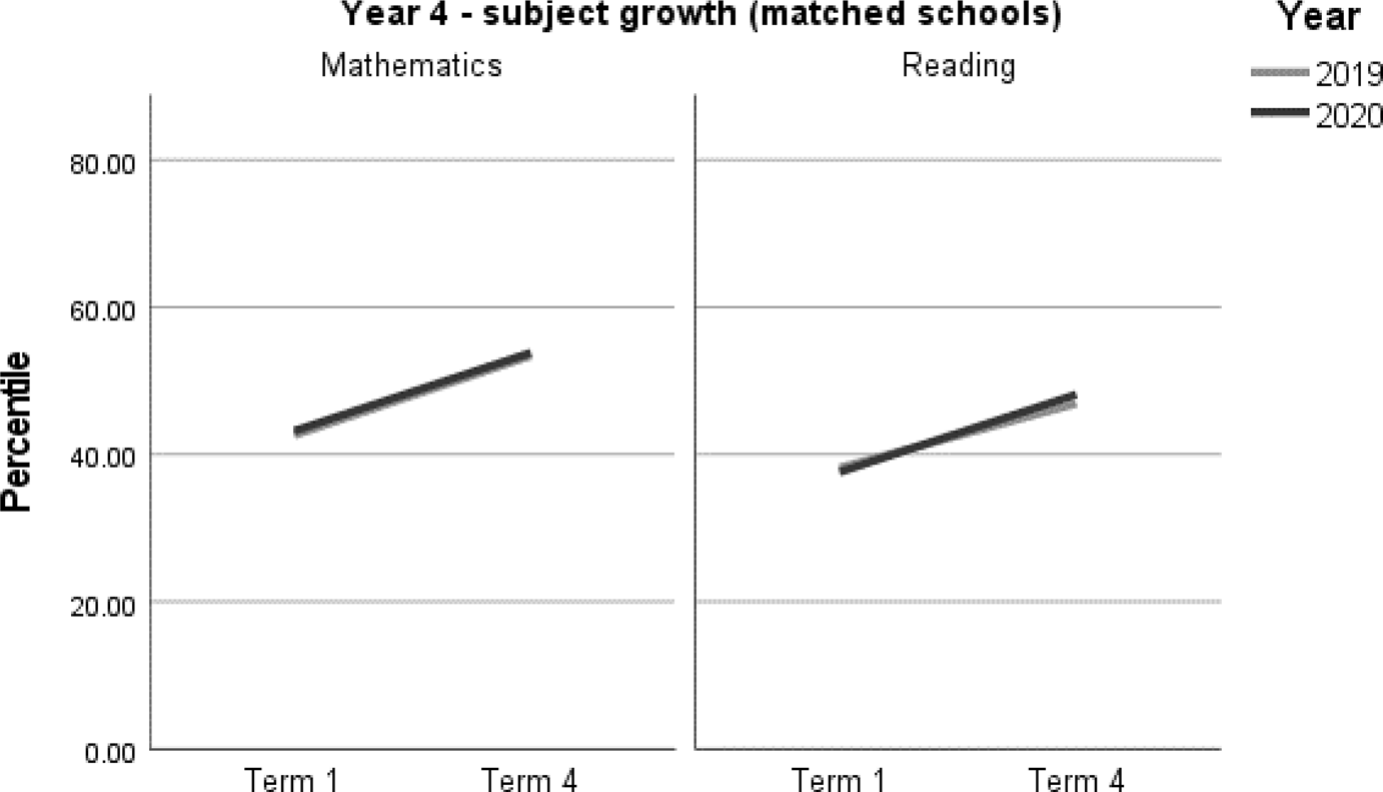
Year 4 student achievement in mathematics and reading (2019–2020)

**Fig. 4:**
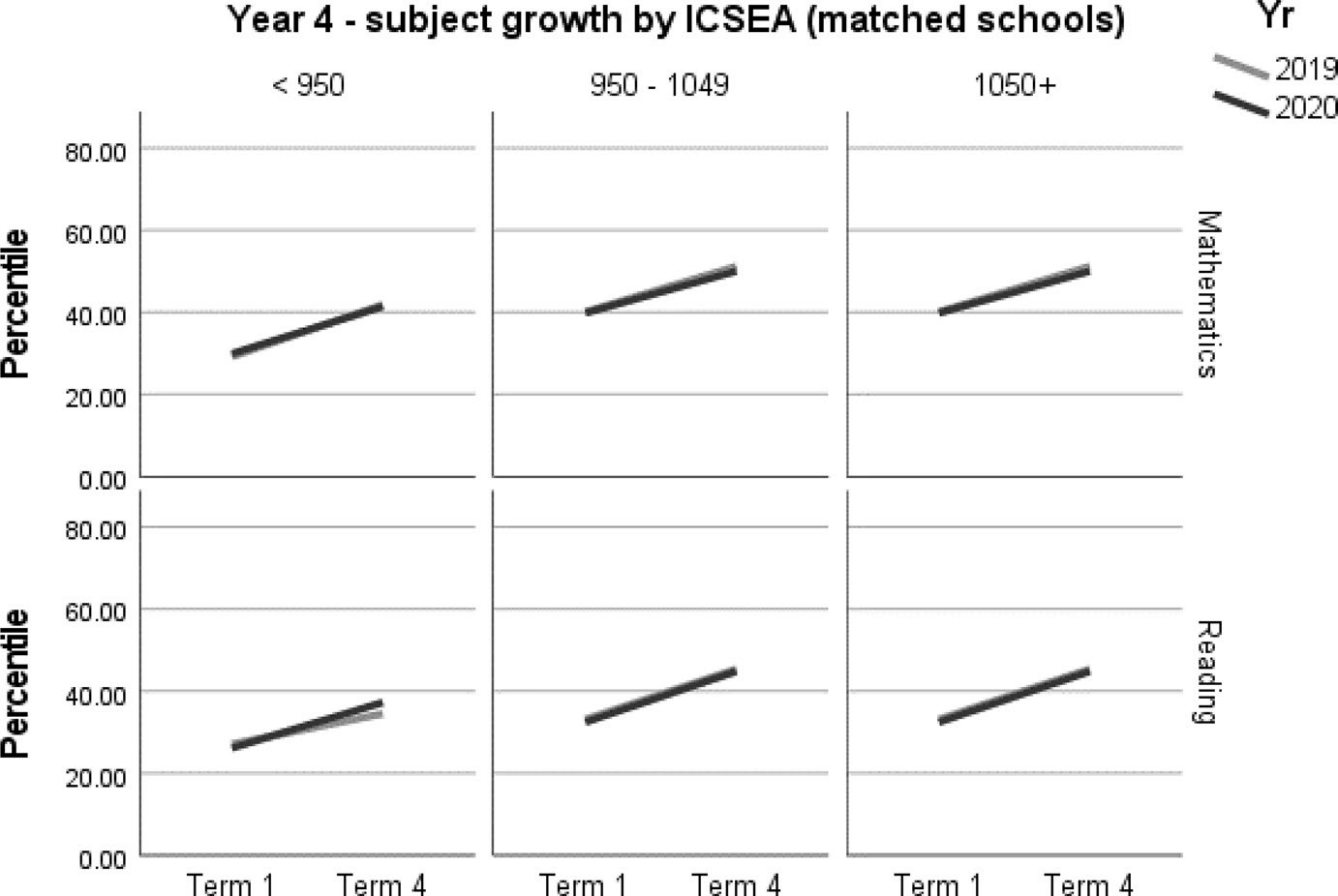
Year 4 student achievement in mathematics and reading (2019–2020) by ICSEA

A summary of student achievement growth in mathematics and reading by ICSEA is displayed in Table 3. For each of the Year 3 and Year 4 samples, no differences in student achievement growth were recorded between 2019 and 2020. However, a more nuanced picture emerged when taking school ICSEA into account. Year 3 students in low-ICSEA schools (ICESA < 950) achieved significantly less growth, equivalent to two months, in mathematics relative to the 2019 cohort (*d* = -0.16; 95% CI = -0.31, -0.01; *p* = 0.033^1^) (Table 5, Fig. 2). In 2020, Year 3 students from schools in the middle ICSEA band (950–1050), achieved the equivalent of two months’ additional growth in mathematics compared with those in the same ICSEA band in 2019 (*d* = 0.15; 95% CI = 0.06, 0.25; *p* = 0.002) (Table 5, Fig. 2). No other significant differences between students in 2019 and 2020 were recorded in mathematics or reading achievement by Year level or by ICSEA (Tables 4, 5, 6, 7, Figs. 1, 2, 3, 4).

**Table 3:** Year 3 and Year 4 student achievement growth in mathematics and reading (2019–2020) by ICSEA

Year	ICSEA	Mathematics	Reading
Year 3	Low	− 2 months	–
	Mid	+ 2 months	–
	High	–	–
	Whole sample	–	–
Year 4	Low	–	–
	Mid	–	–
	High	–	–
	Whole sample	–	–

– denotes no significant difference between the 2019 and 2020 cohorts

The details of these analyses are provided below. First, the overall findings for Year 3 in mathematics and reading are provided, followed by the analysis of ICSEA bands. This pattern is repeated for Year 4. Next, we turn to specific sub-samples of students for whom achievement levels are notoriously, on average, low, and for whom grave concern has been expressed during the pandemic; namely, those in regional locations and Indigenous students.

**Table 4:** Year 3 student achievement in mathematics and reading (2019–2020)

Outcome	*n*	Baseline mean (95% CI)	Ceiling *n* (%)	Retest %	*n* (miss)	Mean change from baseline (95% CI)	Adjusted mean difference (95% CI) ^a^	Adjusted effect size *d* (95% CI) ^a^	*P*
*Year 3*									
*Mathematics*									
2020	670	39.68 (36.4, 42.97)	0 (0)	91	608 (62)	17.20* (15.84, 18.56)	1.65 (− 0.21, 3.52)	0.06 (− 0.01, 0.13)	0.082
2019	757	40.23 (37.02, 43.44)	5 (0.6)	92	693 (64)	15.55* (14.27, 16.82)	Reference	Reference	
*Reading*									
2020	664	30.45 (26.94, 33.97)	3 (0.4)	91	605 (59)	22.67* (21.1, 24.24)	1.15 (− 1, 3.29)	0.04 (− 0.03, 0.11)	0.295
2019	765	29.26 (25.84, 32.68)	0 (0)	91	698 (67)	21.52* (20.06, 22.98)	Reference	Reference	

*CI* Confidence interval

^*^Significance at *p* < 0.05

^a^Between year difference of change score (2020 change minus 2019 change)

**Table 5:** Year 3 student achievement in mathematics and reading (2019–2020) by ICSEA

Outcome	*n*	Baseline mean (95% CI)	Ceiling *n* (%)	Retest %	*n* (miss)	Mean change from baseline (95% CI)	Adjusted mean difference (95% CI)^a^	Adjusted effect size *d* (95% CI)^a^	*p*
*Year 3*									
*Mathematics*									
*ICSEA < 950*									
2020	144	32.5 (27.78, 37.22)	0 (0)	86	124 (20)	11.66* (8.83, 14.49)	− 4.03 (− 7.74, − 0.32)	− 0.16 (− 0.31, − 0.01)	0.033*
2019	190	31.77 (27.47, 36.06)	1 (0.5)	91	173 (17)	15.69* (13.29, 18.09)	Reference	Reference	
*ICSEA 950–1049*									
2020	414	39.19 (35.58, 42.81)	0 (0)	91	375 (39)	18.23* (16.46, 20.01)	4.06 (1.53, 6.59)	0.15 (0.06, 0.25)	0.002*
2019	399	40.04 (36.39, 43.69)	0 (0)	90	360 (39)	14.17* (12.36, 15.98)	Reference	Reference	
*ICSEA 1050 + *									
2020	112	51.92 (46.83, 57.02)	0 (0)	97	109 (3)	19.83* (16.71, 22.95)	1.31 (− 2.74, 5.36)	0.05 (− 0.11, 0.21)	0.525
2019	168	52.78 (48.25, 57.31)	4 (2.2)	95	160 (8)	18.52* (15.95, 21.1)	Reference	Reference	
*Reading*									
*ICSEA < 950*									
2020	148	24.39 (20.05, 28.74)	0 (0)	85	126 (22)	18.41* (14.95, 21.87)	− 1.32 (− 5.88, 3.24)	− 0.05 (− 0.22, 0.12)	0.569
2019	193	20.98 (17.11, 24.86)	0 (0)	89	172 (21)	19.74* (16.77, 22.71)	Reference	Reference	
*ICSEA 950–1049*									
2020	401	28.58 (24.84, 32.31)	3 (0.7)	92	369 (32)	23.70* (21.69, 25.71)	2.21 (− 0.64, 5.07)	0.08 (− 0.02, 0.18)	0.129
2019	397	27.67 (23.94, 31.4)	0 (0)	91	361 (36)	21.49* (19.46, 23.52)	Reference	Reference	
*ICSEA 1050 + *									
2020	115	46.34 (40.64, 52.05)	0 (0)	96	110 (5)	23.98* (20.37, 27.59)	0.48 (− 4.19, 5.14)	0.02 (− 0.15, 0.18)	0.841
2019	175	45.26 (40.19, 50.32)	0 (0)	94	165 (10)	23.50* (20.56, 26.45)	Reference	Reference	

*CI* Confidence interval

^*^Significance at *p* < 0.05

^a^Between year difference of change score (2020 change minus 2019 change)

**Table 6:** Year 4 student achievement in mathematics and reading (2019–2020)

Outcome	*n*	Baseline mean (95% CI)	Ceiling *n* (%)	Retest %	*n* (miss)	Mean change from baseline (95% CI)	Adjusted mean difference (95% CI)^a^	Adjusted effect size *d* (95% CI)^a^	*p*
*Year 4*									
*Mathematics*									
2020	730	43.19 (39.57, 46.82)	7 (0.9)	91	662 (68)	10.62* (9.41, 11.83)	− 0.15 (− 1.84, 1.53)	− 0.01 (− 0.07, 0.06)	0.857
2019	768	42.63 (39.03, 46.23)	6 (0.7)	92	706 (62)	10.78* (9.61, 11.95)	Reference	Reference	
*Reading*									
*Year 4*									
2020	722	37.61 (34.01, 41.2)	2 (0.3)	89	645 (77)	10.53* (9.08, 11.98)	1.8 (− 0.18, 3.79)	0.07 (− 0.01, 0.14)	0.075
2019	793	38.19 (34.66, 41.71)	2 (0.2)	94	742 (51)	8.73* (7.38, 10.09)	Reference	Reference	

*CI* Confidence interval

^a^Between year difference of change score (2020 change minus 2019 change)

^*^Significance at *p* < 0.05

**Table 7:** Year 4 student achievement in mathematics and reading (2019–2020) by ICSEA

Outcome	*n*	Baseline mean (95% CI)	Ceiling *n* (%)	Retest %	*n* (miss)	Mean change from baseline (95% CI)	Adjusted mean difference (95% CI)^a^	Adjusted effect size *d* (95% CI)^a^	*p*
*Year 4*									
*Mathematics*									
*ICSEA < 950*									
2020	148	29.91 (24.62, 35.2)	0 (0)	84	125 (23)	11.49* (8.96, 14.02)	− 1.17 (− 5, 2.65)	− 0.05 (− 0.22, 0.12)	0.545
2019	108	29.2 (23.64, 34.75)	0 (0)	92	99 (9)	12.66* (9.80, 15.52)	Reference	Reference	
*ICSEA 950–1049*									
2020	320	39.83 (36.1, 43.56)	0 (0)	92	293 (27)	10.18* (8.36, 12.01)	− 0.90 (− 3.41, 1.61)	− 0.04 (− 0.14, 0.07)	0.483
2019	358	40.09 (36.48, 43.70)	0 (0)	91	327 (31)	11.08* (9.36, 12.81)	Reference	Reference	
*ICSEA 1050 + *									
2020	262	56.13 (52.28, 59.98)	7 (2.6)	93	244 (18)	10.67* (8.61, 12.72)	0.90 (− 1.91, 3.72)	0.04 (− 0.08, 0.15)	0.528
2019	302	55.33 (51.63, 59.04)	6 (1.9)	93	280 (22)	9.76* (7.84, 11.68)	Reference	Reference	
*Reading*									
*ICSEA < 950*									
2020	138	26.15 (20.71, 31.59)	0 (0)	84	116 (22)	11.11* (7.94, 14.28)	3.68 (− 0.79, 8.15)	0.15 (− 0.03, 0.34)	0.106
2019	124	27.09 (21.74, 32.45)	0 (0)	97	120 (4)	7.43* (4.28, 10.58)	Reference	Reference	
*ICSEA 950–1049*									
2020	315	32.46 (28.94, 35.98)	0 (0)	90	283 (32)	12.37* (10.20, 14.54)	0.11 (− 2.85, 3.06)	0.00 (− 0.11, 0.12)	0.944
2019	359	33.11 (29.74, 36.49)	0 (0)	92	332 (27)	12.27* (10.26, 14.27)	Reference	Reference	
*ICSEA 1050+*									
2020	269	51.76 (48.1, 55.42)	2 (0.7)	91	246 (23)	8.12* (5.73, 10.51)	2.87 (− 0.38, 6.12)	0.11 (− 0.01, 0.23)	0.084
2019	310	53.49 (50, 56.97)	2 (0.6)	94	290 (20)	5.25* (3.05, 7.45)	Reference	Reference	

*CI* Confidence interval

*Significance at *p* < 0.05

^a^Between year difference of change score (2020 change minus 2019 change)

### Student achievement by location

**Fig. 5:**
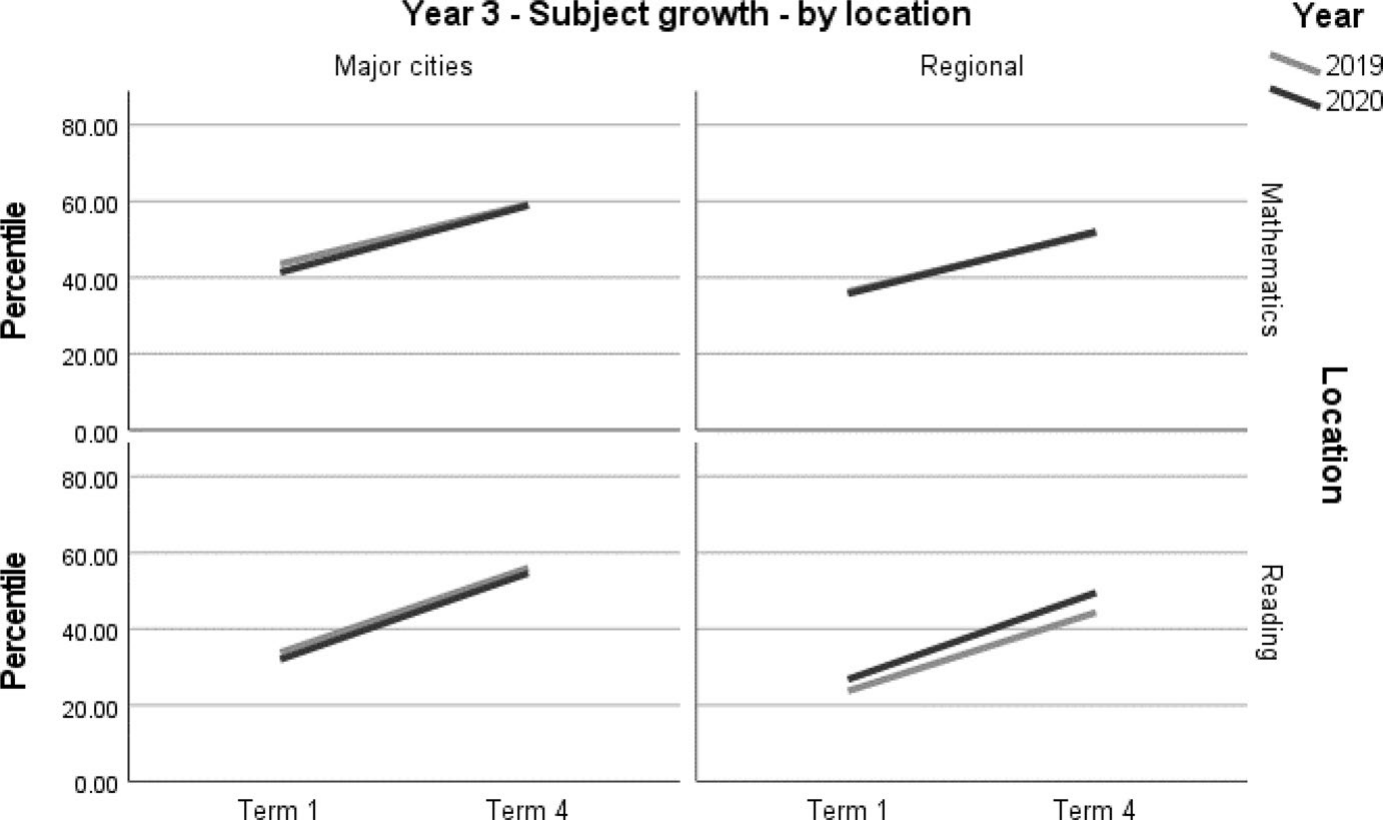
Year 3 student achievement in mathematics and reading (2019–2020) by location

**Fig. 6:**
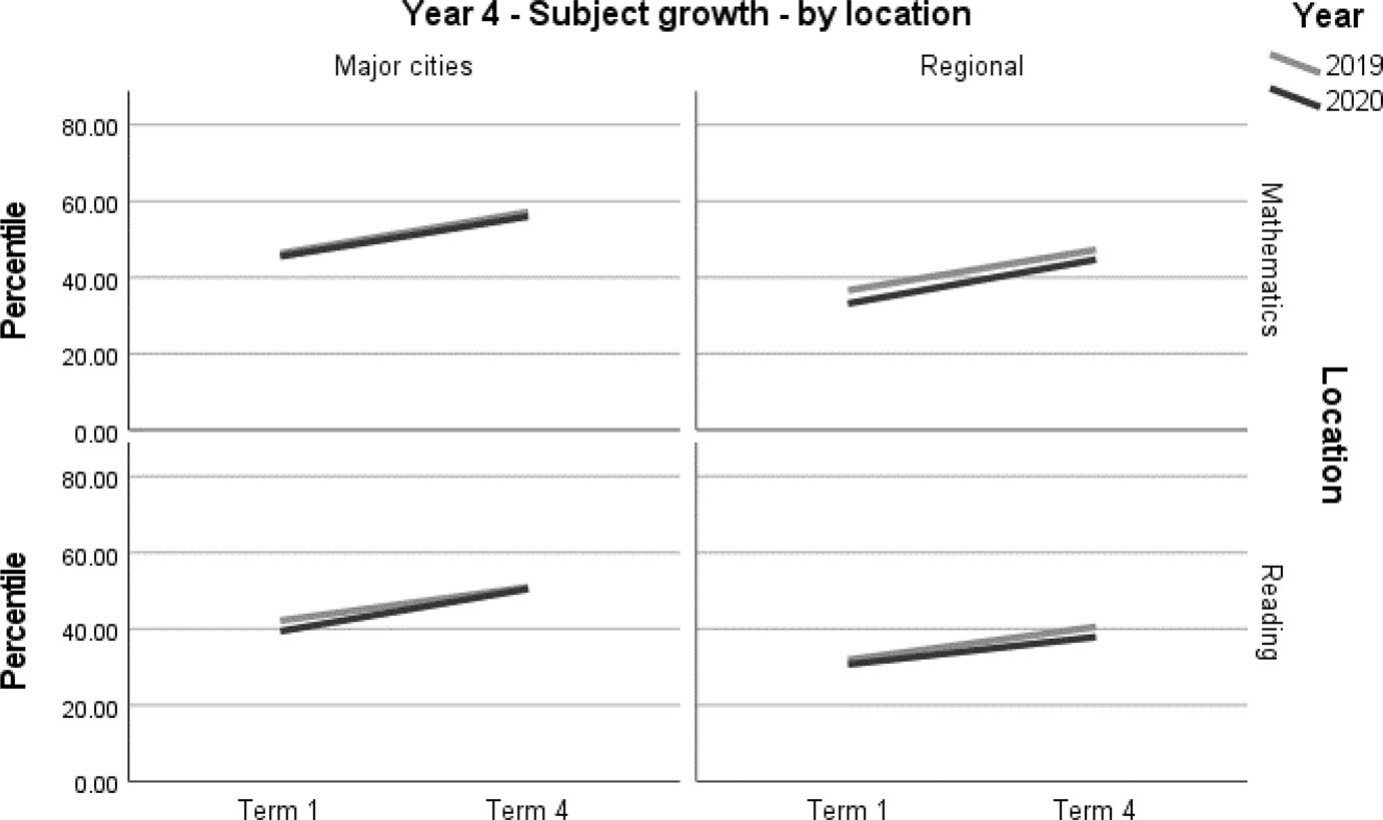
Year 4 regional student achievement in mathematics and reading (2019–2020) by location

A summary of achievement growth in mathematics and reading for students in regional locations and major cities is displayed in Table 8. Students in major cities demonstrated one month’s additional growth (*d* = 0.08; 95% CI = 0.00, 0.17; *p* = 0.047^2^) in reading (Table 9, Figs. 5 and 6). There were no significant differences in mathematics (Table 9, Figs. 5 and 6). Due to the relatively small samples used in this analysis, and the fact that ‘regional’ was defined as outside major cities, these results should be interpreted with caution.

**Table 8:** Student achievement by subject and location (2019–2020)

Year	Location	Mathematics	Reading
3	Major cities	–	–
	Regional	–	–
4	Major cities	–	+ 1 month
	Regional	–	–

– denotes no significant difference between the 2019 and 2020 cohorts

**Table 9:** Year 3 and Year 4 student achievement in mathematics and reading by location (2019–2020)

Outcome	*n*	Baseline mean (95% CI) ^a^	Ceiling *n* (%)	Retest %	*n* (miss)	Mean change from baseline (95% CI) ^a^	Adjusted mean difference (95% CI) ^a^	Adjusted effect size *d* (95% CI) ^a^	*p*
*Year 3*									
*Mathematics*									
*Major cities*									
2020	481	58.90 (55.06, 62.74)	0 (0)	93	447 (34)	17.53* (15.9, 19.16)	1.86 (− 0.52, 4.24)	0.07 (− 0.02, 0.15)	0.125
2019	431	59.22 (54.97, 63.47)	4 (0.9)	92	395 (36)	15.67* (13.94, 17.4)	Reference	Reference	
*Regional*									
2020	189	35.77 (30.05, 41.48)	0 (0)	85	161 (28)	16.27* (13.74, 18.79)	0.87 (− 2.26, 4.01)	0.03 (− 0.09, 0.16)	0.585
2019	326	36.31 (31.76, 40.86)	1 (0.3)	91	298 (28)	15.39* (13.53, 17.25)	Reference	Reference	
*Reading*									
*Major cities*									
2020	484	32.04 (27.82, 36.26)	2 (0.4)	94	454 (30)	22.63* (20.79, 24.47)	0.43 (− 2.25, 3.11)	0.01 (− 0.07, 0.10)	0.753
2019	438	33.81 (29.15, 38.47)	0 (0)	92	405 (33)	22.20* (20.26, 24.15)	Reference	Reference	
*Regional*									
2020	180	26.80 (21.40, 32.21)	1 (0.5)	84	151 (29)	22.73* (19.68, 25.78)	2.14 (− 1.63, 5.90)	0.08 (− 0.06, 0.21)	0.265
2019	327	23.74 (19.52, 27.95)	0 (0)	90	293 (34)	20.59* (18.39, 22.80)	Reference	Reference	
*Year 4*									
*Mathematics*									
*Major cities*									
2020	626	45.55 (41.61, 49.48)	7 (1.1)	91	568 (58)	10.48* (9.17, 11.79)	− 0.36 (− 2.26, 1.54)	− 0.01 (− 0.09, 0.06)	0.710
2019	548	46.31 (41.89, 50.74)	6 (1.1)	93	511 (37)	10.84* (9.46, 12.22)	Reference	Reference	
*Regional*									
2020	104	33.19 (26.36, 40.02)	0 (0)	90	94 (10)	11.50* (8.30, 14.71)	0.90 (− 3.00, 4.80)	0.04 (− 0.12, 0.20)	0.649
2019	220	36.64 (31.76, 41.52)	0 (0)	89	195 (25)	10.60* (8.38, 12.82)	Reference	Reference	
*Reading*									
*Major cities*									
2020	621	39.41 (35.44, 43.38)	2 (0.3)	89	554 (67)	11.10* (9.51, 12.7)	2.32 (0.03, 4.61)	0.08 (0.00, 0.17)	0.047*
2019	559	42.17 (37.72, 46.61)	2 (0.4)	94	524 (35)	8.78* (7.14, 10.43)	Reference	Reference	
*Regional*									
2020	101	30.72 (24.15, 37.28)	0 (0)	90	91 (10)	7.17* (3.58, 10.76)	− 1.46 (− 5.73, 2.81)	− 0.06 (− 0.22, 0.11)	0.502
2019	234	31.89 (27.40, 36.38)	0 (0)	93	218 (16)	8.63* (6.31, 10.95)	Reference	Reference	

*CI* Confidence interval

*Significance at *p* < 0.05

^a^Between year difference of change score (2020 change minus 2019 change)

**Fig. 7:**
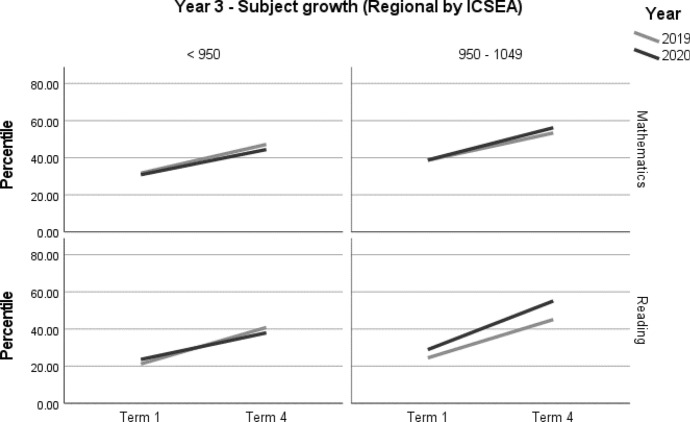
Year 3 regional student achievement in mathematics and reading (2019–2020) by ICSEA

**Fig. 8:**
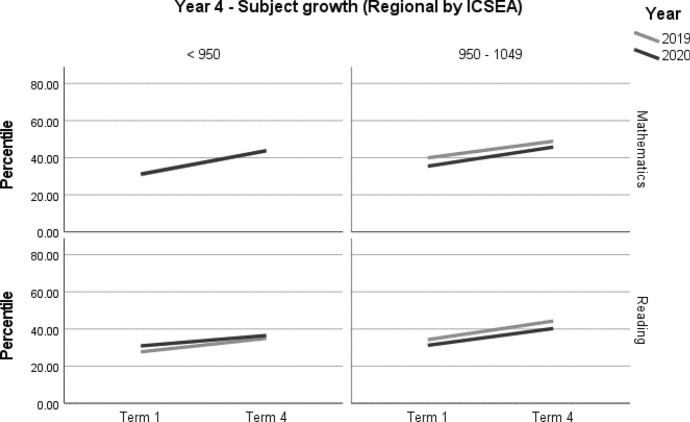
Year 4 regional student achievement in mathematics and reading (2019–2020) by ICSEA

A summary of achievement growth in mathematics and reading for students in regional locations by ICSEA is displayed in Table 10. Year 3 students in mid-ICSEA schools demonstrated three months' additional growth (*d* = 0.20; 95% CI = 0.02, 0.38; *p* = 0.033^3^) in reading (Table 11, Fig. 7). There were no significant differences in mathematics (Table 11, Figs. 7 and 8).

**Table 10:** Regional student achievement by subject and ICSEA (2019–2020)

Year	ICSEA	Mathematics	Reading
3	Low	–	–
	Mid	–	+ 3 months
4	Low	–	–
	Mid	–	–

– denotes no significant difference between the 2019 and 2020 cohorts

**Table 11:** Year 3 and Year 4 regional student achievement (2019–2020) by subject and ICSEA

Outcome	*n*	Baseline mean (95% CI) ^a^	Ceiling *n* (%)	Retest %	*n* (miss)	Mean change from baseline (95% CI) ^a^	Adjusted mean difference (95% CI) ^a^	Adjusted effect size *d* (95% CI) ^a^	*p*	
*Year 3—Regional by ICSEA*										
*Mathematics*										
*< 950*										
2020	58	30.91 (23.46, 38.35)	0 (0)	83	48 (10)	13.54* (9.12, 17.96)	− 1.93 (− 6.97, 3.11)	− 0.08 (− 0.28, 0.13)	0.451	
2019	179	31.71 (26.94, 36.48)	1 (0.5)	91	162 (17)	15.47* (13.05, 17.89)	Reference	Reference		
*950–1049*										
2020	131	38.80 (32.37, 45.22)	0 (0)	86	113 (18)	17.38* (14.22, 20.53)	2.69 (− 1.79, 7.18)	0.11 (− 0.07, 0.29)	0.238	
2019	123	38.66 (32.29, 45.03)	0 (0)	91	112 (11)	14.69* (11.50, 17.87)	Reference	Reference		
*Reading*										
*< 950*										
2020	55	23.62 (16.49, 30.75)	0 (0)	80	44 (11)	14.34* (8.92, 19.77)	− 5.28 (− 11.41, 0.85)	− 0.20 (− 0.43, 0.03)	0.091	
2019	182	21.20 (16.91, 25.50)	0 (0)	88	161 (21)	19.63* (16.77, 22.48)	Reference	Reference		
*950–1049*										
2020	125	28.94 (22.15, 35.73)	1 (0.8)	86	107 (18)	26.18* (22.55, 29.8)	5.57 (0.45, 10.68)	0.20 (0.02, 0.38)	0.033*	
2019	122	24.48 (17.83, 31.13)	0 (0)	89	109 (13)	20.61* (17, 24.21)	Reference	Reference		
*Year 4—Regional by ICSEA*										
*Mathematics*										
*< 950*										
2020	58	43.78 (35.19, 52.37)	0 (0)	88	51 (7)	12.52* (8.60, 16.43)	− 0.40 (− 5.43, 4.64)	− 0.02 (− 0.23, 0.20)	0.877	
2019	87	43.80 (36.92, 50.68)	1 (0.5)	90	78 (9)	12.91* (9.74, 16.08)	Reference	Reference		
*950–1049*										
2020	46	35.47 (25.04, 45.89)	0 (0)	93	43 (3)	10.31* (5.07, 15.54)	1.37 (− 4.87, 7.61)	0.05 (− 0.19, 0.30)	0.665	
2019	116	39.95 (32.82, 47.08)	0 (0)	87	101 (15)	8.94* (5.54, 12.34)	Reference	Reference		
*Reading*										
< 950										
2020	57	30.93 (22.68, 39.18)	0 (0)	86	49 (8)	5.58* (0.82, 10.34)	− 1.69 (− 7.53, 4.14)	− 0.07 (− 0.30, 0.17)	0.567	
2019	103	27.71 (21.56, 33.86)	0 (0)	96	99 (4)	7.28* (3.90, 10.65)	Reference	Reference		
*950–1049*										
2020	44	31.24 (22.77, 39.72)	1 (0.8)	95	42 (2)	9.06* (3.78, 14.33)	− 0.96 (− 7.22, 5.30)	− 0.04 (− 0.27, 0.20)	0.762	
2019	114	34.27 (28.69, 39.84)	0 (0)	90	103 (11)	10.02* (6.66, 13.38)	Reference	Reference		

*CI* Confidence interval

^*^Significance at *p* < 0.05

^a^Between year difference of change score (2020 change minus 2019 change)

### Indigenous student achievement

**Fig. 9:**
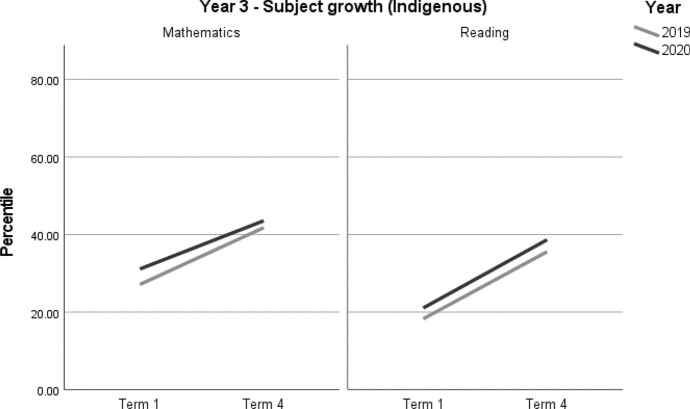
Year 3 Indigenous student achievement in mathematics and reading (2019–2020)

**Fig. 10:**
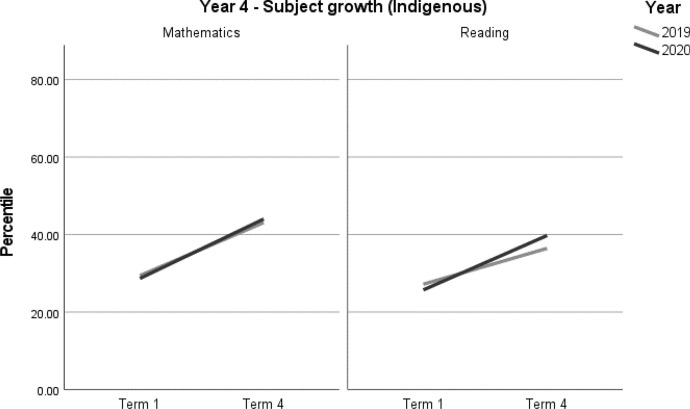
Year 4 Indigenous student achievement in mathematics and reading (2019-2020)

For the Indigenous students in the sample, no differences in achievement growth were recorded between the 2019 and 2020 cohorts, by subject (Table 12, Figs. 9 and 10). Due to the relatively small samples used in this analysis we were unable to analyse Indigenous student achievement by school ICSEA. For this reason, these results should be interpreted with caution (Tables 12, 13) (Fig. 9 and 10).

**Table 12:** Indigenous student achievement in mathematics and reading (2019–2020) by ICSEA

Year	ICSEA	Mathematics	Reading
3	Low	–	–
	Mid	–	–
4	Low	–	–
	Mid	–	–

– denotes no significant difference between the 2019 and 2020 cohorts

**Table 13:** Indigenous student achievement in mathematics and reading (2019–2020)

Outcome	*n*	Baseline mean (95% CI)	Ceiling *n* (%)	Retest %	*n* (miss)	Mean change from baseline (95% CI)	Adjusted mean difference (95% CI)^a^	Adjusted effect size *d* (95% CI)^a^	*p*
*Year 3*									
*Indigenous*									
*Mathematics*									
2020	58	31.13 (23.97, 38.30)	0 (0)	78	45 (13)	12.43* (7.39, 17.47)	− 2.21 (− 8.88, 4.45)	− 0.09 (− 0.36, 0.18)	0.512
2019	69	27.14 (20.47, 33.82)	0 (0)	88	61 (8)	14.65* (10.28, 19.01)	Reference	Reference	
*Reading*									
2020	59	38.67 (31.73, 45.60)	0 (0)	78	46 (13)	17.58* (12.16, 23.01)	0.28 (− 6.92, 7.47)	0.01 (− 0.28, 0.31)	0.939
2019	69	35.59 (29.30, 41.88)	0 (0)	90	62 (7)	17.30* (12.58, 22.03)	Reference	Reference	
*Year 4*									
*Indigenous*									
*Mathematics*									
2020	37	28.69 (19.93, 37.44)	0 (0)	81	30 (7)	15.32* (10.96, 19.69)	1.65 (− 4.77, 8.06)	0.07 (− 0.20, 0.33)	0.610
2019	30	29.42 (20.20, 38.65)	0 (0)	87	26 (4)	13.68* (8.98, 18.38)	Reference	Reference	
*Reading*									
2020	36	39.77 (31.80, 47.74)	0 (0)	81	29 (7)	14.05* (8.50, 19.60)	4.80 (− 3.21, 12.81)	0.20 (− 0.13, 0.53)	0.235
2019	31	36.44 (27.99, 44.90)	0 (0)	87	27 (4)	9.25* (3.48, 15.02)	Reference	Reference	

*CI* Confidence interval

^*^Significance at *p* < 0.05

^a^Between year difference of change score (2020 change minus 2019 change)

### Instructional volume

Teachers reported providing the largest volume of instruction in reading, followed by mathematics (Table 14). Reported time spent in reading for comprehension, as a specific reading focus, was approximately half that of the reported time spent in mathematics instruction, across all groups. Overall, more time was spent on literacy in the 2020 group ($\overline{x}$ = 9.52 h per week) compared to 2019 ($\overline{x}$ = 8.48) and on reading for comprehension in 2020 ($\overline{x}$ = 3.48 h per week) compared to 2019 ($\overline{x}$ = 3.07). Numeracy was reported as receiving more time during 2020 ($\overline{x}$ = 6.76) than in 2019 ($\overline{x}$ = 6.74)—this was particularly true for Term 4 ($\overline{x}$ = 7.04), after the return to schooling.

**Table 14:** Instructional volume (hours per week) literacy, reading and numeracy (2019–2020)

Subject area	Term	2019 h/week	*N*	2020 h/week	*N*
		Mean (SD)		Mean (SD)	
Literacy total	T1			9.27 (2.31)	41
	T3			9.36 (2.52)	39
	T4	8.48 (3.83)		9.87 (3.23)	47
	Total	8.48 (3.83)	27	9.52 (2.74)	127
Reading for comprehension	T1			3.24 (1.61)	41
	T3			3.26 (1.83)	39
	T4	3.07 (1.84)		3.87 (1.95)	47
	Total	3.07 (1.84)	27	3.48 (1.82)	127
	T1			6.46 (1.91)	41
Numeracy	T3			6.74 (2.06)	39
	T4	6.74 (3.84)		7.04 (2.23)	47
	Total	6.74 (3.84)	27	6.76 (2.08)	127

## Discussion

The COVID-19 pandemic disrupted schooling throughout the world on a scale never seen before (UNESCO 2020a). In NSW government schools, the disruption was relatively short; it took the form of an 8–10 week ‘learning from home’ period in which most students engaged in schooling remotely. In this paper, we examined the effects of the COVID-19 pandemic and learning from home on student achievement in mathematics and reading. Effects on student and teacher well-being, which were substantial, will be the focus of separate papers in order to do justice to the important issues raised.

Although ‘learning loss’ is now part of the 2020 lexicon, together with ‘unprecedented’, ‘pivot’ and ‘you’re on mute’, we have deliberately avoided the expression throughout this paper to guard against literal readings and causing undue worry among parents and the wider community. Students learned and achieved during 2020. They did not go backward or lose what they had learned. Rather, some did not achieve the same level of growth as students in the previous cohort. Most affected, according to our analysis, were Year 3 students in lower ICSEA schools in mathematics. We return to these findings shortly.

### The importance of context

Speculation about the impact of COVID-19 and learning from home on student academic achievement has been widespread, relying heavily on evidence and modelling from previous crisis situations. However, the size and scale of disruption caused by COVID-19 is truly unprecedented and cannot directly be compared with these earlier accounts. Our study provides rigorous empirical evidence of what happened to student achievement in Years 3 and 4, in NSW, during the pandemic. While the analysis has implications for countries around the world, we note that extrapolation even within Australia should be approached with care. In the state of Victoria, for example, schools were closed for around 18–20 weeks while schools in the Northern Territory were closed for just four days at the end of Term 1 (Storen and Corrigan 2020). Such contextual differences require vigilance when interpreting research findings.

To date, with the exception of the Dorn et al. (2020) report from the United States and the Engzell et al. (2020) report using data from the Netherlands, we have found no quantitative evidence of the impact of COVID-19 on student academic achievement. Interpreting the results of these (any) studies must take important contextual differences into account. For example, the Dorn et al. (2020) report is based on a secondary analysis of data collected by Curriculum Associates (2020). The data were collected from more than 250,000 students across 28 states in the United States, each with different ‘closedown’ or ‘learning from home’ periods’. In addition, they compare test scores to the average achievement of students in the previous three testing cycles.

The Engzell et al. (2020) analysis shares more similarities with our own, given that both studies are based on data collected before and after an eight-week period of school closure and a relevant comparison group, but the follow-up data in the Netherlands were collected straight after the return to school. Such immediate measures were not possible in our study, given the exclusion of non-essential personnel from schools. Nor did we want to burden teachers or students with additional testing when many were under great stress already.

In our study, students attended school for most of Term 1 and were (mostly) back by Term 3. The follow-up data collection a full term after the return to school therefore represents achievement growth over the entire year, not just during the learning from home period. Before closedown, students and teachers in our study had established relationships and ways of working that would have helped in the shift to learning from home. By contrast, the new school year in the United States, framed by astonishing levels of COVID-19 (at the time of writing, 27.4 million cases and 470,000 deaths) compared with Australia, could be expected to negatively affect student testing. These differences in research design and local circumstances are critical to meaningful comparison of findings.

### Predicted versus actual impact on student learning

While it was broadly predicted that students would face some ‘learning loss’ during the COVID-19 learning from home period (Brown et al. 2020; OECD 2020; Pedro Azevedo et al. 2020; Sawchuk 2020; United Nations 2020), our study indicates that growth in student achievement during the 2020 school year varied minimally from growth in achievement in 2019. This result might partly be accounted for by the relatively short closedown period and by the timing of our achievement growth measures, one term after the return to school for most students.

Reading achievement was not significantly different for either Year 3 or Year 4 students. Additional time spent reading, supported by family members, during the learning from home period may have been a factor in these results. Furthermore, there was no apparent effect on mathematics achievement for Year 4 students. The only significant effects were for Year 3 students in mathematics whereby those in mid-ICSEA schools showed an additional two months’ growth and those in low-ICSEA schools showed two months less growth than the comparison schools.

If students fell behind in their learning during closedown, as the *Check In* assessments in NSW government schools suggested (Baker, 2020), our study indicates that teachers have done an outstanding job in helping students draw level with and even overtake (in the case of students in mid-ICSEA schools in mathematics) expected achievement levels. They have ensured that achievement, at least in maths and reading, is as strong as usual (taking the 2019 cohort to be indicative of student growth in a typical year). Our results also signal the capacity of students to learn despite serious disruption to ‘schooling as usual’. Teacher reports of students’ increased facility with technology as a result of learning from home may have been a factor in the varying achievement growth by ICSEA. Instructional volume might also have contributed to these results. That is, teachers reported spending more time in mathematics and reading during Term 3 and Term 4 than in Term 1, of 2020, and more time than teachers reported in Term 4 of 2019. This increase in subject-specific instructional time is likely to have played a role in students ‘catching up’.

### Concern for the most vulnerable

However, as predicted by many commentators (Brown et al. 2020; Schleicher 2020; Sonnemann and Goss 2020), there were some negative effects on student achievement in lower ICSEA (disadvantaged) schools, particularly for younger students. The lower growth in mathematics for Year 3 students in these schools might be explained by the greater challenges faced by families in disadvantaged circumstances who are likely to have been disproportionately impacted by the pandemic (Institute for Social Science Research [ISSR] 2020). It is worth highlighting that our finding of two months' less growth in mathematics in less advantaged schools was associated with a remote learning period of around two months. In contexts where schools were closed for much longer periods (such as in Victoria, the United States and many European nations), research is urgently needed to understand and ameliorate the effects of COVID-19 on the learning of vulnerable students.

The results we obtained for students in regional locations, which follow a similar pattern of extra growth for students in mid-ICSEA schools are noteworthy but less robust given the smaller samples. Stories we heard from teachers of some country kids spending the learning from home period working and playing on the family farm may have been a factor for some.

The result of no significant differences for Indigenous students between 2019 and 2020 is cause for celebration, given that lower growth might have been predicted given, on average, their over-representation in lower ICSEA schools. It is a testament to their families and teachers that no negative effects of COVID-19 and learning from home were evident in their academic achievement. On the other hand, achievement levels for Indigenous students in Australia have consistently been significantly below those of their non-Indigenous peers which means there is still much to do in working towards more equitable outcomes.

In all disadvantaged contexts, ameliorating lower growth in academic achievement is likely to require significant investment in the form of additional support for teachers and students. The recently announced $ 337 million tutoring scheme (NSW Government 2020) has a critical role to play here. It represents a unique opportunity to address longstanding inequities as well as those exacerbated by the pandemic, if done well (Slavin 2020).

## Conclusion

Given limited system-level data globally on the effects of COVID-19 on student learning, partly because of the pandemic’s timing relative to the school year in the northern hemisphere and partly because of limited access to directly comparable data, this study offers unique insight based on rigorous evidence. This study’s significance lies in demonstrating that, in NSW at least, the disruptions to schooling caused by COVID-19 did not have the kinds of dire consequences for student learning that many commentators had predicted. Although specific to NSW, these findings are likely to resonate across Australia and across the globe given our shared experience in this (hopefully) once-in-a lifetime occurrence.

Despite even well-informed speculation on the potential effects of COVID-19 on student learning (see for example, Baker 2020; Hargreaves and Fullan 2020; Henebery 2020; Joseph and Fahey 2020), very little of this commentary is grounded in empirical evidence. Drawing on directly comparable data from 2019, our study provides clear evidence of the impacts of the COVID-19 pandemic on schooling in 2020 in one state school system. These results provide an important counter-narrative to widespread speculation about alarming levels of ‘learning loss’. While the lower achievement growth in mathematics for Year 3 students in lower ICSEA schools must be addressed, urgently if existing inequities are not to be further entrenched, most students are, academically, where they are expected to be. Our findings are a testament to the dedicated work of teachers during 2020 to ensure that learning for most students was not compromised despite unusually trying circumstances. School systems elsewhere in Australia and around the world may find this evidence helpful in establishing a solid empirical basis for investigating what happened to student learning during COVID-19, in their own contexts.

## Appendix

See Table

**Table 15:** Sample characteristics—whole sample, (2019, 2020)

Characteristics	Total		Year 3		Year 4	
	2019	2020	2019	2020	2019	2020
Schools, *n*	62	51	57	44	56	48
ICSEA^a^, mean (SD) ^b^	995 (82)	1007 (76)	987 (80)	999 (72)	991 (79)	1009 (77)
ICSEA < 950, *n* (%)	19 (31)	10 (20)	19 (33)	9 (20)	18 (32)	9 (19)
ICSEA 950–1049, *n* (%)	29 (47)	25 (49)	27 (47)	24 (55)	27 (48)	23 (48)
ICSEA 1050 +, *n* (%)	14 (23)	16 (31)	11 (19)	11 (25)	11 (20)	16 (33)
Rural, *n* (%)	27 (44)	11 (22)	27 (47)	11 (25)	24 (42)	10 (20)
Major cities	35 (56)	40 (78)	30 (53)	33 (75)	32 (57)	38 (79)
Inner regional	21 (34)	10 (20)	21 (37)	10 (23)	18 (32)	9 (19)
Outer regional	5 (8)	1 (2)	5 (9)	1 (2)	5 (9)	1 (2)
Remote	0 (0)	0 (0)	0 (0)	0 (0)	0 (0)	0 (0)
Very remote	1 (2)	0 (0)	1 (2)	0 (0)	1 (2)	0 (0)
Students, *n*	2738	2156	1332	1016	1406	1140
Age—years, mean (SD)	9.7 (0.7)	9.7 (0.7)	9.2 (0.4)	9.1 (0.5)	10.1 (0.4)	10.1 (0.4)
Female, *n* (%)	1354 (50)	1073 (50)	657 (49)	510 (50)	697 (50)	563 (50)
Indigenous, *n* (%)	185 (7)	132 (6)	111 (8)	74 (7)	74 (5)	58 (5)
LBOTE, *n* (%)	666 (24)	522 (24)	306 (23)	248 (24)	360 (26)	274 (24)

15.

## Footnotes

1 While this result is significant using a traditional *p* value of < 0.05, it is no longer significant using a Bonferroni adjusted alpha of < 0.025 to account for two primary outcomes—achievement growth in reading and mathematics.

2 While this result is significant using a traditional *p* value of < 0.05, it is no longer significant using a Bonferroni adjusted alpha of < 0.025 to account for two primary outcomes—achievement growth in reading and mathematics.

3 While this result is significant using a traditional *p* value of < 0.05, it is no longer significant using a Bonferroni adjusted alpha of < 0.025 to account for two primary outcomes—achievement growth in reading and mathematics.
